# Numerical Issues for Solving Eu-type Generalized Hydrodynamic Equations to Investigate Continuum-rarefied Gas Flows

**DOI:** 10.1038/s41598-018-36431-y

**Published:** 2019-01-22

**Authors:** Hong Xiao, Qijiao He, Di Wu

**Affiliations:** 1 School of Power and Energy, Northwestern Polytechnical University, Xi’an, 710072 China; 2 Department of Pure Mathematics and Mathematical Statistics, University of Cambridge, Cambridge, CB3 0WA UK; 3 Department of Mechanical Engineering, The Hong Kong Polytechnic University, Hung Hom, Kowloon, Hong Kong China

## Abstract

Eu-type generalized hydrodynamic equations have been derived from the Boltzmann kinetic theory and applied to investigate continuum and/or rarefied gas flows. This short communication first reports detailed and important issues in the use of the mixed discontinuous Galerkin method to solve Eu-type generalized hydrodynamic equations in multidimensions. Three major issues are reported. These include the treatment of solid boundary conditions for the nonlinear constitutive equations, a slope limiter to maintain high accuracy and avoid unphysical oscillations, and the computational efficiency compared with that of the particle method. In addition, we implement the present model to a rigid problem, which includes gas flows around the NACA0018 airfoil, a sharp wedge, a sphere and a three-dimensional Apollo configuration.

## Introduction

The numerical studies on nonequilibrium gas flows are of certain interest because this research provides fundamental knowledge of flow physics^1–3^, *et al*. It is generally accepted that under non-near equilibrium conditions, such as for non-continuum or rarefied gases in space or in a micro-channel, the Navier-Stokes-Fourier (NSF) theory has very serious limitations in describing the correct flow physics^4,5^. Much effort has been put forth to develop computational frameworks beyond the classical NSF theory^6–8^. These work include two categories: kinetic models and fluid dynamical models. The fluid dynamical models are constructed on the basis of the conservation laws and the constitutive equations of non-conserved variables, and the latter can be derived from Boltzmann kinetic theory. The fluid dynamical models may be classified into a few classes: Eu’s generalized hydrodynamic equations^9,10^, Burnett equations and Grad’s moment model. These fluid dynamical models were derived from the Boltzmann kinetic theory, with an emphasis on reducing the computational cost. A significant result of Eu’s generalized hydrodynamic equations is that the constitutive equations of non-conserved variables, such as heat flux and stresses, were obtained from the Boltzmann kinetic theory by considering entropy conditions. Then, the constitutive equations were simplified by omitting the high-order term and unsteady term then named as the nonlinear coupled constitutive relations (NCCR)^11^. Later, the unsteady term was considered by the present author^12^. Since the works of NCCR and our previous work are both based on Eu’s generalized hydrodynamic equations, we will call these methods Eu-type Equations to differentiate this study from the previous work^13^.

A mixed discontinuous Galerkin (DG) method has been given to solve Eu-type Equations^12^. However, doubts still remain over the numerical algorithms for the implementation of solid boundary conditions and the limiter. Moreover, the computational efficiency and the case study of the heat flux in hypersonic gas flows were not performed. In this regards, the aim of this study is to fill these gaps.

## Eu-Type Generalized Hydrodynamic Equations

Curtiss introduced the Boltzmann equation for a rigid diatomic molecule by adding an angular momentum *J* and a moment of inertia *I*. It can be written, without external field, as

1 $$[\frac{\partial }{\partial t}+{\bf{v}}\cdot \nabla +\frac{J}{I}\frac{\partial }{\partial \psi }]\,f({\bf{v}},{\bf{r}},t)=R[\,f].$$

Here, *R*[*f*] denotes collision term which account for the interactions with other particles. The equation 1 is irreversible and thus expected to give a description of macroscopic processes that progress irreversibly towards equilibrium. Most importantly, this characteristic is a more useful form due to the entropy conditions.

The three quantities of mass, momentum and energy are conserved variables and their molecular expressions are collisional invariants in the irreversibly progress towards equilibrium. Therefore, collision term do not contribute to the conservation equations. Then, the three conservation equations can be easily derived from Boltzmann kinetic theory by differentiating the three statistical formulas with time and then substituting them into equation 1. However, the molecular expressions of nonconserved variables can not yield the collisional invariant. These nonconserved variables include the heat flux **Q**, the shear stress **Π** and the excess normal stress Δ. They are usually referred as nonconserved moments. We may use the differential equation of velocity moment to obtain the constitutive equations. This procedure contains two significant issues: the treatment of collision term and the distribution function definition. In the procedure of deriving Eu-type generalized hydrodynamic equations, the treatment of collision term is determined by the dissipation nonlinear cumulant approximation. And, distribution function evolves as macroscopic moments function. Most importantly, the entropy production strictly dictates the flux dependence of distribution function. And the previous works^9^ have described the detailed procedure. Finally, Eu-equations can be written as:

2 $$\rho \tfrac{D}{Dt}[\begin{array}{l}{[1,\rho {\bf{u}},\rho E]}^{T}/\rho \\ {\boldsymbol{\Pi }}/\rho \\ ({\boldsymbol{\Pi }}+{\rm{\Delta }}\delta )/\rho \\ {\bf{Q}}/\rho \end{array}]+\nabla \cdot [\begin{array}{l}{[{\bf{u}},{\boldsymbol{\Pi }}+(p+{\rm{\Delta }})\delta ,({\boldsymbol{\Pi }}+(p+{\rm{\Delta }})\delta )\cdot {\bf{u}}+{\bf{Q}}]}^{T}\\ {{\boldsymbol{\Psi }}}^{({\boldsymbol{\Pi }})}\\ {{\boldsymbol{\Psi }}}^{({\rm{\Delta }})}\\ {{\boldsymbol{\Psi }}}^{({\bf{Q}})}\end{array}]=[\begin{array}{l}{[0,0,0]}^{T}\\ {Z}^{({\boldsymbol{\Pi }})}+{{\rm{\Lambda }}}^{({\boldsymbol{\Pi }})}\\ {Z}^{({\rm{\Delta }})}+{{\rm{\Lambda }}}^{({\rm{\Delta }})}\\ {Z}^{({\bf{Q}})}+{{\rm{\Lambda }}}^{({\bf{Q}})}\end{array}]$$

In Eq. 2 the conservation variables of mass density, fluid velocity, and total energy density, denoted by *ρ*, **u** and *E*, are similar to those of the NSF framework. Moreover, *p* and *T* denote the pressure and gas temperature, respectively. In addition to the conservation variables, the non-conserved variables of the high-order flux term, the kinematic term and the dissipative term, represented by ***Ψ***, *Z* and Λ, are expressed as

$$\begin{array}{rcl}{{\boldsymbol{\Psi }}}^{({\boldsymbol{\Pi }})} & = & \langle m{[{\bf{c}}{\bf{c}}]}^{(2)}{\bf{c}}[\,f]\rangle ,\\ {{\boldsymbol{\Psi }}}^{(\Delta )} & = & \langle (\frac{1}{3}m{c}^{2}-\frac{p}{n}){\bf{c}}[\,f]\rangle ,\\ {{\boldsymbol{\Psi }}}^{({\boldsymbol{Q}})} & = & \langle (\frac{1}{2}m{c}^{2}+{H}_{rot}-m\hat{h}){\bf{c}}{\bf{c}}[f]\rangle ,\\ {Z}^{({\boldsymbol{\Pi }})} & = & \langle f(\frac{D}{Dt}+{\bf{c}}\cdot {\boldsymbol{\nabla }}+\frac{j}{I}\frac{\partial }{\partial \Psi })m{[{\bf{c}}{\bf{c}}]}^{(2)}\rangle ,\\ {Z}^{(\Delta )} & = & \langle f(\frac{D}{Dt}+{\bf{c}}\cdot {\boldsymbol{\nabla }}+\frac{j}{I}\frac{\partial }{\partial \Psi })(\frac{1}{3}m{c}^{2}-\frac{p}{n})\rangle ,\\ {Z}^{({\boldsymbol{Q}})} & = & \langle f(\frac{D}{Dt}+{\bf{c}}\cdot {\boldsymbol{\nabla }}+\frac{j}{I}\frac{\partial }{\partial \Psi })(\frac{1}{2}m{c}^{2}+{H}_{rot}-m\hat{h}){\bf{c}}\rangle \\ {\Lambda }^{({\boldsymbol{\Pi }})} & = & \langle m{[{\bf{c}}{\bf{c}}]}^{(2)}R[f]\rangle ,\\ {\Lambda }^{(\Delta )} & = & \langle (\frac{1}{3}m{c}^{2}-\frac{p}{n})R[\,f]\rangle ,\\ {\Lambda }^{({\boldsymbol{Q}})} & = & \langle (\frac{1}{2}m{c}^{2}+{H}_{rot}-m\hat{h}){\bf{c}}R[\,f]\rangle \mathrm{.}\end{array}$$

Here, *m* and **c** denote the molecular mass and the peculiar velocity, respectively. Angular brackets $\langle \cdots \rangle =\int \,{\rm{d}}{\bf{v}}\cdots$ represents the integration over velocity space. ${[]}^{(2)}$ stands for the symmetric traceless part of the second rank tensor. We imposed physically motivated conditions on the closure of high order terms: $\nabla \cdot {\boldsymbol{\Psi }}()$ change faster than the non-conserved evaluation. Thus, they can be ignored. Eu approach^9,10^ is imposed to close *Z* and Λ.

3 $$\begin{array}{l}{Z}^{({\boldsymbol{\Pi }})}=\frac{{{\boldsymbol{\Pi }}}_{0}}{\eta /p}-\mathrm{2[}{\boldsymbol{\Pi }}\cdot \nabla {\bf{u}}{]}^{\mathrm{(2)}},{Z}^{({\rm{\Delta }})}=\frac{3{{\rm{\Delta }}}_{0}}{2\gamma ^{\prime} {\eta }_{b}/p}-2\gamma ^{\prime} ({\boldsymbol{\Pi }}+{\rm{\Delta }}\delta ):\,\nabla {\bf{u}}\\ {Z}^{({\bf{Q}})}=(1+\frac{{\boldsymbol{\Pi }}}{p})\cdot \frac{{{\bf{Q}}}_{0}}{\lambda /p{C}_{p}}+\frac{1}{Pr}\frac{{\bf{Q}}}{\lambda /p{C}_{p}}\cdot \frac{(\,-\,\eta \nabla {\bf{u}})}{p}\end{array}$$

4 $$\begin{array}{l}{{\rm{\Lambda }}}^{({\boldsymbol{\Pi }})}=-\,\frac{{\boldsymbol{\Pi }}}{\eta /p}q(\kappa ),{{\rm{\Lambda }}}^{({\rm{\Delta }})}=-\,\frac{{\rm{\Delta }}{\bf{I}}}{{\eta }_{b}/p}q(\kappa )\\ {{\rm{\Lambda }}}^{({\bf{Q}})}=\frac{{\bf{Q}}}{\lambda /p{C}_{p}}q(\kappa )\end{array}$$

Equation 2 can be further normalized by the reference variables and rewritten in a more compact form as^9,10^,

5 $$\{\begin{array}{l}\frac{\partial }{\partial t}{\bf{U}}+\nabla \cdot {{\bf{F}}}_{{\rm{inv}}}({\bf{U}})+\nabla \cdot {{\bf{F}}}_{{\rm{inv}}}({\bf{S}})=\mathrm{0,}\\ \frac{\partial }{\partial t}{\bf{S}}+\nabla \cdot {\bf{G}}({\bf{S}})+\nabla \cdot {\bf{H}}({\bf{S}})=0.\end{array}$$

Here,

6 $${\bf{U}}=(\begin{array}{c}\rho \\ \rho {\bf{u}}\\ \rho E\end{array}),{{\bf{F}}}_{{\rm{inv}}}=(\begin{array}{c}\rho {\bf{u}}\\ \rho {\bf{u}}{\bf{u}}+\tfrac{p}{\gamma M{a}^{2}}{\boldsymbol{\delta }}\\ (\rho E+\tfrac{p}{\gamma M{a}^{2}}){\bf{u}}\end{array}),{{\bf{F}}}_{{\rm{vis}}}=\tfrac{1}{{Re}}(\begin{array}{c}0\\ {\boldsymbol{\Pi }}+{\rm{\Delta }}\delta \\ ({\boldsymbol{\Pi }}+{\rm{\Delta }}\delta )\cdot {\bf{u}}+\tfrac{1}{(\gamma -\mathrm{1)}M{a}^{2}Pr}{\bf{Q}}\end{array})$$

7 $$\begin{array}{rcl}{\bf{S}} & = & (\begin{array}{c}\hat{{\boldsymbol{\Pi }}}\\ \hat{{\boldsymbol{\Pi }}}+{f}_{b}\hat{{\rm{\Delta }}}\delta \\ \hat{{\bf{Q}}}\end{array}),{\bf{G}}({\bf{S}})=(\begin{array}{c}\hat{{\boldsymbol{\Pi }}}{\hat{{\boldsymbol{\Pi }}}}_{0}\\ (\hat{{\boldsymbol{\Pi }}}+{f}_{b}\hat{{\rm{\Delta }}}\delta ){\hat{{\boldsymbol{\Pi }}}}_{0}\\ \hat{{\bf{Q}}}{\hat{{\boldsymbol{\Pi }}}}_{0}\end{array}),\\ {\bf{H}}({\bf{S}}) & = & (\begin{array}{c}\mathrm{2(1}+{f}_{b}\hat{{\rm{\Delta }}}){\hat{{\boldsymbol{\Pi }}}}_{0}+\mathrm{2[}\hat{{\boldsymbol{\Pi }}}\cdot {\hat{{\boldsymbol{\Pi }}}}_{0}{]}^{\mathrm{(2)}}-2\hat{{\boldsymbol{\Pi }}}q(c\hat{R})\\ 2\gamma ^{\prime} (\hat{{\boldsymbol{\Pi }}}+{f}_{b}\hat{{\rm{\Delta }}}\delta )\,:\,{\hat{{\boldsymbol{\Pi }}}}_{0}+\frac{4\gamma ^{\prime} {f}_{b}}{3}{\hat{{\rm{\Delta }}}}_{0}+\frac{4\gamma ^{\prime} {f}_{b}}{3}\hat{{\rm{\Delta }}}q(c\hat{R})\\ 2Pr\mathrm{(1}+{f}_{b}\hat{{\rm{\Delta }}}){\hat{{\bf{Q}}}}_{0}+2Pr{\hat{{\bf{Q}}}}_{0}\cdot \hat{{\boldsymbol{\Pi }}}+\hat{{\bf{Q}}}\cdot {\hat{{\boldsymbol{\Pi }}}}_{0}-2Pr\hat{{\bf{Q}}}q(c\hat{R})\end{array})\end{array}$$

Here, subscript 0 stands for the linear constitutive relations in the NSF framework: Fourier law and Newtonian law. *γ* is the specific heat and *γ*′ = (5 − 3*γ*)/2. *f*_*b*_ = *η*_*b*_/*η*, where *η*_*b*_ is bulk viscosity and *η* is shear viscosity. *δ* is the unit tensor. *Re*, *Pr* and *Ma* are dimensionless parameters of gas dynamics, i.e., the Reynolds, Prandtl and Mach numbers, respectively.

8 $$\begin{array}{c}\hat{{\boldsymbol{\Pi }}}=\frac{{N}_{\delta }}{p}{\boldsymbol{\Pi }},\hat{{\rm{\Delta }}}=\frac{{N}_{\delta }}{p}{\rm{\Delta }},\hat{{\bf{Q}}}=\frac{{N}_{\delta }}{p}\frac{{\bf{Q}}}{\sqrt{T[(\gamma -\mathrm{1)}PrM{a}^{2}\mathrm{]/2}}},\\ {N}_{\delta }=\frac{\gamma M{a}^{2}}{Re},{\hat{R}}^{2}=\hat{{\boldsymbol{\Pi }}}\,:\,\hat{{\boldsymbol{\Pi }}}+2{f}_{b}\gamma ^{\prime} \hat{{\rm{\Delta }}}+\hat{{\bf{Q}}}\cdot \hat{{\bf{Q}}},q(\kappa )\equiv \frac{sinh\kappa }{\kappa }\mathrm{.}\end{array}$$

9 $${{\boldsymbol{\Pi }}}_{0}=-\,2\eta {[{\bf{u}}]}^{\mathrm{(2)}},{{\rm{\Delta }}}_{0}=-\,{\eta }_{b}\nabla {\bf{u}},{{\bf{Q}}}_{0}=-\,\lambda \nabla T$$

We chose the mixed DG algorithm as the solver in this study. The DG algorithm achieves high-accuracy solutions by using a local higher-order approximation. This treatment is substantially different from conventional FVM and FDM in which a high accuracy is obtained by using wide stencils. The numerical algorithm for the DG method for solving Eu-type equations in this study was fully discussed and validated in our previous work. Therefore, only a brief review will be given here.

For implementing the mixed DG, the Eu-type equations (Eq. 5) can be expressed in conservation form^14^ as

10 $$\{\begin{array}{l}\frac{\partial }{\partial t}\,{\int }_{{\rm{\Omega }}}\,{{\bf{U}}}_{h}\phi {\rm{d}}V+{\int }_{{\rm{\Gamma }}}\,\phi {{\bf{F}}}_{{\rm{inv}}}{\rm{d}}s-{\int }_{{\rm{\Omega }}}\,\nabla \phi {{\bf{F}}}_{{\rm{inv}}}{\rm{d}}V+{\int }_{{\rm{\Gamma }}}\,\phi {{\bf{F}}}_{{\rm{vis}}}{\rm{d}}s=0-{\int }_{{\rm{\Omega }}}\,\nabla \phi {{\bf{F}}}_{{\rm{vis}}}{\rm{d}}V,\\ \frac{\partial }{\partial t}\,{\int }_{{\rm{\Omega }}}\,{{\bf{S}}}_{h}\phi {\rm{d}}V-{\int }_{{\rm{\Gamma }}}\,\phi {\bf{G}}({{\bf{S}}}_{h}){\rm{d}}s+{\int }_{{\rm{\Omega }}}\,[\nabla \phi {\bf{G}}({{\bf{S}}}_{h})+\phi {\bf{H}}({{\bf{S}}}_{h})]{\rm{d}}V=0.\end{array}$$

Here, **U**_*h*_ and **S**_*h*_ are numerically approximations of the solutions for **U** and **S** in the local cell of Ω. Γ represents the cell interfaces Ω. **U**_*h*_ and **S**_*h*_ can be further expressed by their higher order approximations,

$${{\bf{U}}}_{h}({\bf{x}},t)=\sum _{i=1}^{N}\,{U}^{i}(t){\phi }_{i}({\bf{x}}),{{\bf{S}}}_{h}({\bf{x}},t)=\sum _{i=1}^{N}\,{S}^{i}(t){\phi }_{i}({\bf{x}})$$

where *φ* denotes the basis function. And, the basis number *N* is determined by the approximation order.

The equations for **S** in Eq. 10 were first solved to compute the non-conservation variables after **U**(**x**, *t*) was updated during each iteration step.

For the inviscid terms in Eq. 10, the local Lax-Friedrichs (LxF) flux^13,15^, **h**_inv_, was applied to estimate the flux through each element boundary as

11 $${{\bf{h}}}_{{\rm{inv}}}({{\bf{U}}}^{+},{{\bf{U}}}^{-},{\bf{n}})=\frac{1}{2}[{{\bf{F}}}_{{\rm{inv}}}({{\bf{U}}}^{-})+{{\bf{F}}}_{{\rm{inv}}}({{\bf{U}}}^{+})-{C}_{{\rm{inv}}}({{\bf{U}}}^{+}-{{\bf{U}}}^{-})]$$

where

$${C}_{{\rm{inv}}}=max(|{u}^{+}|+{c}_{s}^{+}/Ma,|{u}^{-}|+{c}_{s}^{-}/Ma)$$

and ${c}_{s}=\sqrt{T}$ represents the speed of sound at the cell interface. The positive and negative signs represent the normal directions towards the outsides and insides of the cell interface.

We applied Zhang positivity-preserving flux^16,17^ for the viscous term **h**_vis_.

12 $$\begin{array}{rcl}{{\bf{h}}}_{{\rm{vis}}}({{\bf{U}}}^{+},{{\bf{S}}}^{+},{{\bf{U}}}^{-},{{\bf{S}}}^{-};{\bf{n}}) & \cong  & {\int }_{{\rm{\Gamma }}}\,{\boldsymbol{\phi }}{{\bf{F}}}_{{\rm{vis}}}{\rm{d}}x\\  & = & \frac{1}{2}[{{\bf{F}}}_{{\rm{vis}}}({{\bf{U}}}^{+},{{\bf{S}}}^{+})+{{\bf{F}}}_{{\rm{vis}}}({{\bf{U}}}^{-},{{\bf{S}}}^{-})-{C}_{{\rm{vis}}}({{\bf{U}}}^{+}-{{\bf{U}}}^{-})]\end{array}$$

where

$$\begin{array}{rcl}{C}_{{\rm{vis}}} & = & MAX({\beta }^{-},{\beta }^{+}),\\ \beta  & = & MAX\tfrac{1}{2\rho eRe(\gamma -\mathrm{1)}M{a}^{2}Pr}(\sqrt{|{\bf{Q}}\cdot {\bf{n}}{|}^{2}+(\gamma -\mathrm{1)}M{a}^{2}Pr2e\parallel {\boldsymbol{\Pi }}\cdot {\bf{n}}{\parallel }^{2}}+|{\bf{Q}}\cdot {\bf{n}}|),\\ \rho e & = & \frac{p}{\gamma (\gamma -\mathrm{1)}M{a}^{2}}\end{array}$$

The central flux can be used for the **S** terms

13 $${{\bf{h}}}_{G,H}({{\bf{S}}}^{-},{{\bf{S}}}^{+};{\bf{n}})\cong {\int }_{{\rm{\Gamma }}}\,{\boldsymbol{\phi }}{\bf{G}}({{\bf{S}}}_{h}){\rm{d}}s=\frac{1}{2}[{{\bf{G}}}^{-}+{{\bf{G}}}^{+}]$$

Notice that the Gaussian quadrature is applied to estimate the volume integrals in the element Ω. Subsequently, Eq. 10 can be rewritten in a semi-discrete form as

14 $${\bf{L}}\frac{\partial {\bf{S}}}{\partial t}={{\bf{R}}}_{S}({\bf{S}}),$$

15 $${\bf{L}}\frac{\partial {\bf{U}}}{\partial t}={{\bf{R}}}_{U}({\bf{U}},{\bf{S}})$$

By applying the multi-order Runge-Kutta scheme, Eq. 14 can be solved.

### Treatment of the boundary conditions

The mixed DG scheme for Eu-type generalized hydrodynamic equations can be conducted via the following steps:

1. Computing the ${\hat{{\bf{Q}}}}_{0}$, ${\hat{{\rm{\Delta }}}}_{0}$ and ${\hat{{\boldsymbol{\Pi }}}}_{0}$ based on a gradient of the approximation of the conserved variables, **U**_**h**_.
2. Computing **H(S**_**h**_**)** and **G(S**_**h**_**)** in Eq. (10).
3. Computing the integration and flux of the evolution equations for **S**_**h**_ through the Gaussian quadrature in Eq. (10). Updating the approximation of **S**_**h**_ via the Runge-Kutta method.
4. Based on the updated approximation of **S**_**h**_, updating **Q**, Δ and **Π** in the evolution equations for **U**_**h**_. Updating the approximation of **U**_**h**_ via the Runge-Kutta method.
5. Returning to step (1) until the convergent error is satisfied.

The conventional boundary conditions in the CFD, such as the Dirichlet boundary conditions, can be implemented to solve Eu-type equations in the mixed DG framework. Most importantly, the setting of viscous stress on the solid boundary can be avoided.

### Limiter

**Figure 1:**
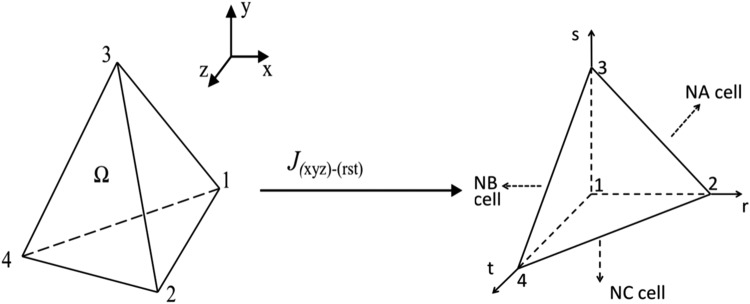
Transform between the arbitrary tetrahedral (*x*, *y*, *z*) and the transferred standard tetrahedral (*r*, *s*, *t*) element.

A coupled slope limiter was employed. For a *P*^2^ expansion, the approximate solution, *U*_*h*_, in three dimensions (see Fig. 1) can be expressed in the arbitrary tetrahedral (*x*, *y*, *z*) or in the transferred standard tetrahedral (*r*, *s*, *t*) element as

16 $${U}_{h}=\sum _{i=1}^{10}\,{U}^{i}{\phi }_{i}(x,y,z)=\sum _{i=1}^{10}\,{U}^{i}{\phi }_{i}(r,s,t)$$

The mean value of *U*_*h*_ and the i-th derivative of the different order approximations are found in the reference standard tetrahedral element, *T*, as shown in Fig. 1.

17 $$\begin{array}{rcl}{\overline{U}}_{h} & = & {U}^{1},\,\begin{array}{rcl}\overline{\frac{\partial {U}_{h}}{\partial r}} & = & 12{U}^{2}-6{U}^{6}-18{U}^{8},\end{array}\\ \overline{\frac{\partial {U}_{h}}{\partial s}} & = & 6{U}^{2}+18{U}^{3}+{U}^{5}+{U}^{6}-2{U}^{7}+{U}^{8}+3{U}^{9}\end{array}$$

18 $$\begin{array}{rcl}\overline{\frac{\partial {U}_{h}}{\partial t}} & = & 6{U}^{2}+6{U}^{3}+24{U}^{4}+{U}^{5}+{U}^{6}+{U}^{7}+{U}^{8}+{U}^{9}-5{U}^{10},\\ \overline{\frac{{\partial }^{2}{U}_{h}}{\partial r\partial s}} & = & 36{U}^{5}+60{U}^{6}\end{array}$$

19 $$\begin{array}{rcl}\overline{\frac{{\partial }^{2}{U}_{h}}{\partial r\partial t}} & = & 36{U}^{5}+12{U}^{6}+72{U}^{8},\\ \overline{\frac{{\partial }^{2}{U}_{h}}{\partial s\partial t}} & = & 12{U}^{5}+36{U}^{6}+48{U}^{7}+36{U}^{8}+108{U}^{9}\end{array}$$

20 $$\begin{array}{rcl}\overline{\frac{{\partial }^{2}{U}_{h}}{\partial {r}^{2}}} & = & 72{U}^{5},\,\begin{array}{rcl}\overline{\frac{{\partial }^{2}{U}_{h}}{\partial {s}^{2}}} & = & 12{U}^{5}+60{U}^{6}+120{U}^{7},\end{array}\\ \overline{\frac{{\partial }^{2}{U}_{h}}{\partial {t}^{2}}} & = & 12{U}^{5}+12{U}^{6}+12{U}^{7}+72{U}^{8}+72{U}^{9}+180{U}^{10}\end{array}$$

Given a tetrahedral element, *T*, its standard triangle neighbors are denoted by NA, NB and NC. We apply the same concept in which the i-th derivative of the numerical solution should not exceed forward and backward differences of the (i-1)-th derivative during the construction of the limiting process. Thus, for the local element in Fig. 1, we arrive at the following system:

21 $$\begin{array}{c}12{\tilde{U}}^{2}-6{\tilde{U}}^{6}-18{\tilde{U}}^{8}=\tilde{\overline{\frac{\partial {U}_{h}}{\partial r}}}=rminmod(\overline{\frac{\partial {U}_{h}}{\partial r}},\frac{1}{2}({\overline{U}}_{h}-{\overline{U}}_{h,NB}))\end{array}$$

22 $$\begin{array}{c}6{\tilde{U}}^{2}+18{\tilde{U}}^{3}+{\tilde{U}}^{5}+{\tilde{U}}^{6}-2{\tilde{U}}^{7}+{\tilde{U}}^{8}+3{\tilde{U}}^{9}\,=\,\tilde{\overline{\tfrac{\partial {U}_{h}}{\partial s}}}\\ \,=\,rminmod(\overline{\tfrac{\partial {U}_{h}}{\partial s}},\tfrac{1}{2}({\overline{U}}_{h}-{\overline{U}}_{h,NC}))\end{array}$$

23 $$\begin{array}{c}6{\tilde{U}}^{2}+6{\tilde{U}}^{3}+24{\tilde{U}}^{4}+{\tilde{U}}^{5}+{\tilde{U}}^{6}+{\tilde{U}}^{7}+{\tilde{U}}^{8}+{\tilde{U}}^{9}-5{\tilde{U}}^{10}\,=\,\tilde{\overline{\frac{\partial {U}_{h}}{\partial t}}}\\ \,=\,r{minmod}(\overline{\frac{\partial {U}_{h}}{\partial t}},\frac{1}{2}({\overline{U}}_{h}-{\overline{U}}_{h,NA}))\end{array}$$

24 $$36{\tilde{U}}^{5}+60{\tilde{U}}^{6}=\tilde{\overline{\frac{{\partial }^{2}{U}_{h}}{\partial r\partial s}}}=rminmod(\overline{\frac{{\partial }^{2}{U}_{h}}{\partial r\partial s}},\frac{1}{2}(\overline{\frac{\partial {U}_{h}}{\partial r}}-{(\overline{\frac{\partial {U}_{h}}{\partial r}})}_{NC}))$$

25 $$\begin{array}{c}36{\tilde{U}}^{5}+12{\tilde{U}}^{6}+72{\tilde{U}}^{8}=\tilde{\overline{\frac{{\partial }^{2}{U}_{h}}{\partial r\partial t}}}=rminmod(\overline{\frac{{\partial }^{2}{U}_{h}}{\partial r\partial t}},\frac{1}{2}(\overline{\frac{\partial {U}_{h}}{\partial r}}-{(\overline{\frac{\partial {U}_{h}}{\partial r}})}_{NA}))\end{array}$$

26 $$\begin{array}{c}12{\tilde{U}}^{5}+36{\tilde{U}}^{6}+48{\tilde{U}}^{7}+36{\tilde{U}}^{8}+108{\tilde{U}}^{9}\,=\,\tilde{\overline{\frac{{\partial }^{2}{U}_{h}}{\partial s\partial t}}}\\ \,=\,rminmod(\overline{\frac{{\partial }^{2}{U}_{h}}{\partial s\partial t}},\frac{1}{2}(\overline{\frac{\partial {U}_{h}}{\partial s}}-{(\overline{\frac{\partial {U}_{h}}{\partial s}})}_{NA}))\end{array}$$

27 $$72{\tilde{U}}^{5}=\tilde{\overline{\frac{{\partial }^{2}{U}_{h}}{\partial {r}^{2}}}}=rminmod(\overline{\frac{{\partial }^{2}{U}_{h}}{\partial {r}^{2}}},\frac{1}{2}(\overline{\frac{\partial {U}_{h}}{\partial r}}-{(\overline{\frac{\partial {U}_{h}}{\partial r}})}_{NB}))$$

28 $$\begin{array}{rcl}12{\tilde{U}}^{5}+60{\tilde{U}}^{6}+120{\tilde{U}}^{7} & = & \tilde{\overline{\frac{{\partial }^{2}{U}_{h}}{\partial {s}^{2}}}}\\  & = & rminmod(\overline{\frac{{\partial }^{2}{U}_{h}}{\partial {s}^{2}}},\frac{1}{2}(\overline{\frac{\partial {U}_{h}}{\partial s}}-{(\overline{\frac{\partial {U}_{h}}{\partial s}})}_{NC}))\end{array}$$

29 $$\begin{array}{c}12{\tilde{U}}^{5}+12{\tilde{U}}^{6}+12{\tilde{U}}^{7}+72{\tilde{U}}^{8}+72{\tilde{U}}^{9}+180{\tilde{U}}^{10}\,=\,\tilde{\overline{\frac{{\partial }^{2}{U}_{h}}{\partial {t}^{2}}}}\\ \,=\,{rminmod}(\overline{\frac{{\partial }^{2}{U}_{h}}{\partial {t}^{2}}},\frac{1}{2}(\overline{\frac{\partial {U}_{h}}{\partial t}}-{(\overline{\frac{\partial {U}_{h}}{\partial t}})}_{NA}))\end{array}$$

The i-th derivative of the numerical solutions of the local elements in Eqs (21–29) are obtained by Eqs (17–20). Finally, ${\tilde{U}}^{i}(i=2\ldots 10)$ can be obtained by solving Eqs (21–29). Thus, *U*_*h*_ is approximated by

30 $${U}_{h}={U}^{1}{\phi }_{1}+\sum _{i=2}^{10}\,{\tilde{U}}^{i}{\phi }_{i}$$

## Results

### NACA0018 results

**Figure 2:**
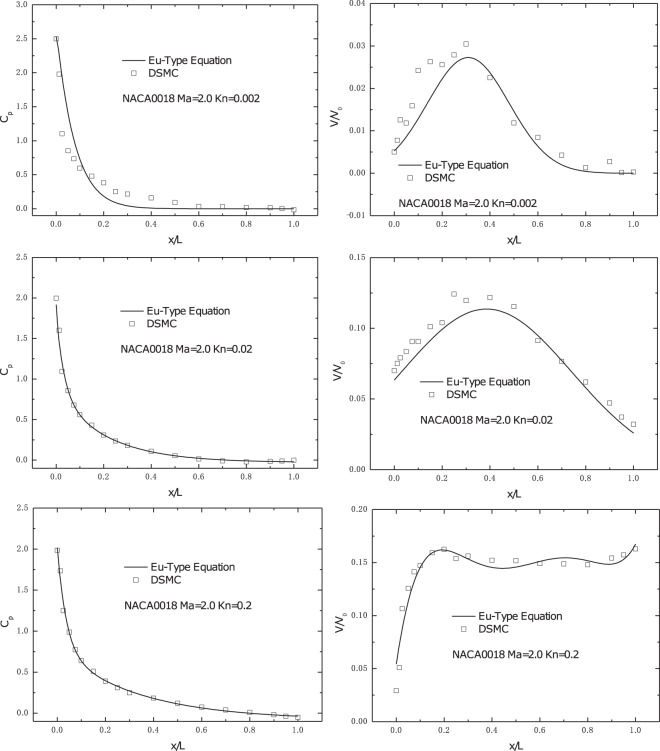
Pressure coefficients and slip velocity on the solid wall for different *Kn* numbers (*Kn* = 0.002, 0.02, 0.2) at **Ma** = **2.0**.

Comparisons of the pressure coefficient, *c*_*p*_, and slip velocity between the Eu-type equations solution and DSMC solution are shown in Fig. 2. The flow condition is *Ma* = 2.0 and *Kn* = 0.002, 0.02, 0.2, respectively, with 0° for AOA. Because of the existing shock wave, a noticeable pressure peak can be found at the leading edge of the airfoil. Thus, a drastic decrease was observed, as shown in Fig. 2. Additionally, the Eu-type equations results show well agreement with the DSMC solution. Similar to the tendency of the pressure coefficient, the maximum value of the slip velocity is at the leading edge of the airfoil. However, in contrast to the tendency of *c*_*p*_, the slip velocity maintains a plateau in most part of the airfoil. The comparison with the DSMC solution shows that there is good agreement in the slip velocity distribution, suggesting that our simulation is reliable.

### Sharp wedge results

**Figure 3:**
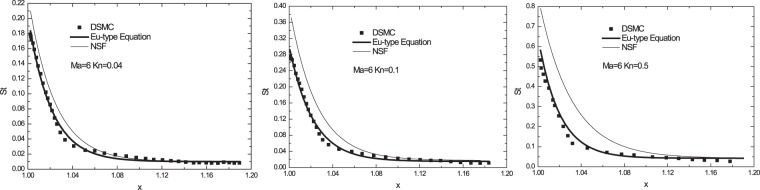
Heat flux on the solid wall of a sharp wedge for different *Kn* numbers (*Kn* = 0.04, 0.1, 0.5).

As the second case study, we gave the results of the flow around a sharp wedge at Mach 6^18^. The half wedge angle was 8°. The Knudsen numbers based on the diameter were 0.04, 0.1 and 0.5. We placed the sharp wedge in the centre of a 30*r* × 30*r* (*r* denotes the head radius) circle calculated domain. After the convergence error test, 500 nodes and 200 nodes were placed in the radial and in the wall directions, respectively. We applied the far-field boundary on the outside circle, and the Langmuir boundary on the solid wall, repetitively. The solid wall temperature was set to be 500 K. Figure 3 shows the solid wall heat flux values on the sharp wedge as estimated by the Eu-type equations and DSMC, which are in well agreement for all three considered cases.

### The gas flow around a 3D sphere

**Figure 4:**
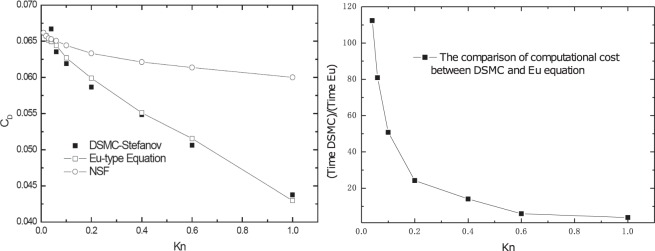
Normalized drag coefficient and computational cost versus Knudsen number at **Re** = **0.125**.

As the third case study, we gave the results of the gas flow around a 3D sphere from *Kn* = 0.01 to *Kn* = 1.0 at *Re* = 0.125^12^. As shown in Fig. 4, we compared the drag coefficient, *C*_*D*_, with the Knudsen number. The calculation results for DG-NSF overpredicted the drag coefficients at all high Knudsen numbers. However, when Kn approached zero, the DG-NSF solutions converged to the DG-Eu and DSMC solution. Barber^19^ also observed these results. When the Knudsen numbers are high, the DG-EU simulations are in well agreement with the DSMC simulations. Additionally, whether the continuum hypothesis holds is indicated by the Knudsen number in the gas flows. The continuum regime has small Knudsen numbers, whereas the rarefied gas flow has high values. Normally, solving the NSF for continuum gas flows can describe one part of the simulated domain. However, for the other part, the particle density is so low that this part is usually simulated by DSMC and can be described as rarefied flows.

The ratio of computational time required between the DCMC and DG-Eu calculations, defined as $\frac{Tim{e}_{DSMC}}{Tim{e}_{DG-Eu}}$, is shown in Fig. 4. It is apparent that the DG-Eu calculation has a much higher computational efficiency than the DSMC method, particularly in regimes near the continuum states. When the *Kn* is less than 0.2, the ratio of computational cost was over 20 times, which implies that even in a rarefied flow, say $Kn\geqslant 0.6$, the computational cost of a DG-Eu calculation is still lower than the cost of DSMC. Therefore, Eu-type generalized hydrodynamic equations provide a better method for considering the entire flow regime.

### A hypersonic gas flow around the Apollo 6 Command Module

The present DG-Eu scheme is also extended to a more complex problem in our previous study^12^: gas flows around the three-dimensional Apollo 6 command module^20^. We placed the Apollo 6 command module in the centre of a 30*d* × 30*d* × 30*d* sphere calculation domain, where *d* denotes the maximum diameter. Then, 5,229 triangular elements were placed on a solid surface, and calculation domain consisted of a total of 1,525,230 cells. When the surface properties changed by less than 10^−7^, the solutions were regarded as convergent. We assumed the surface temperature equalled to the free stream temperature and was uniformly distributed at a constant value. The Knudsen numbers are based on a characteristic length of 3.912 m (maximum capsule diameter) and free stream conditions.

**Figure 5:**
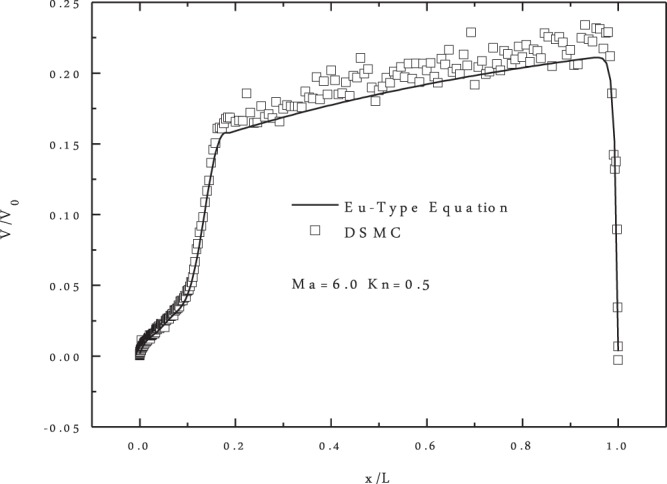
Slip velocity on the solid wall at **Ma** = **6.0** and **Kn** = **0.5**.

**Figure 6:**
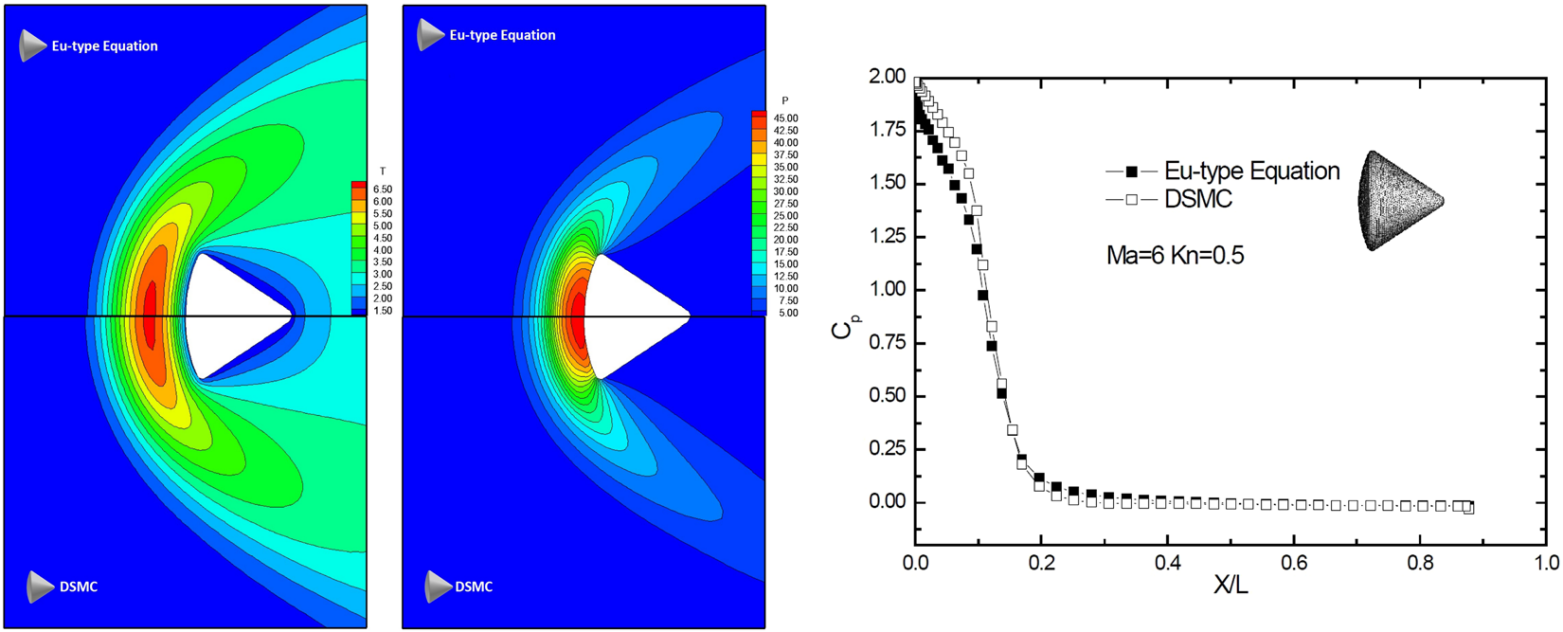
Temperature and pressure contours and pressure coefficients on the solid wall at **Ma** = **6.0** and **Kn** = **0.5**.

The slip velocity distribution according to the DG-Eu solution was first compared with the DSMC solution, as shown in Fig. 5. The DSMC simulation was implemented by the Bird open software^21^, and the slip velocity from both solutions was kept in a trough at the leading edge. This is mainly because of the blunt geometry of the Apollo model. After the blunt leading edge, a rapid increase occurred in $0.2\geqslant x\geqslant 0.1$. In comparison with the DSMC solution, there was good agreement between the DG-Eu and DSMC solutions. The maximum difference was only 0.025, which suggests that the DG-Eu solution can capture the features of a steady flow field. Further comparisons of the pressure and temperature coefficients are plotted in Fig. 6. A well agreement was also found, except a slight difference in the temperature distributions between the DSMC and DG-Eu results. Additionally, we compared the pressure coefficient on the solid wall for DG-EU and DG-NSF at *Ma* = 6.0 and *Kn* = 0.5, as shown in Fig. 6. The results were in well agreement as expected.

## Discussion

Much effort has been put forth to to develop computational frameworks beyond the classical NSF theory to investigate continuum-rarefied gas flows. The Eu-type equations represent one of the fluid dynamical models developed from the Boltzmann equation with an emphasis on reducing the computational cost.

A mixed DG framework was provided to solve the Eu-type equations in our previous studies. However, questions still remain regarding the numerical algorithms for solid boundary conditions and the limiter. Moreover, the computational efficiency and a case study of the heat flux in hypersonic gas flows were not performed. This short note fills these gaps.

We applied our present numerical algorithm to the rigid problems of hypersonic gas flows. It shows that the yield solutions of the Eu-type equations are in well agreement with benchmark data. The results show that this is a successful scheme for solving Eu-type equations, providing a unified framework to model rarefied and continuum gas flows.
